# Risk factors for visual impairment and blindness amongst black adult diabetics receiving treatment at Government healthcare facilities in Mopani District, Limpopo province, South Africa

**DOI:** 10.4102/phcfm.v6i1.623

**Published:** 2014-11-21

**Authors:** Raymond G. Mabaso, Olalekan A. Oduntan

**Affiliations:** 1 Department of Health Studies, University of South Africa, South Africa; 2 Discipline of Optometry, University of KwaZulu-Natal, South Africa

## Abstract

**Background:**

Diabetes mellitus (DM) is a common systemic disease amongst Black South Africans. It may lead to diabetic retinopathy (DR), a common cause of visual impairment (VI) and blindness. DR may significantly increase the prevalence of VI and blindness.

**Aim:**

To assess risk factors for VI and blindness amongst a black diabetic South African population aged ≥ 40 years.

**Setting:**

The study was conducted in seven Government healthcare facilities (two hospitals, four clinics and one health centre) in Mopani District, Limpopo province, South Africa.

**Methods:**

This was a cross-sectional health facility-based quantitative study. Structured interviews were used to obtain information, which included sociodemographic profile, knowledge about DM and its ocular complications, presence of hypertension and accessibility to health facilities. Subsequently participants were examined for VI and blindness using an autorefractor, pinhole disc, ophthalmoscope and logMAR visual acuity chart. Anthropometric measurements (height, weight and waist) were also taken. Associations between 31 risk factors and VI as well as blindness were statistically examined.

**Results:**

Participants (*N* = 225) included 161 women and 64 men aged 40–90 years (mean 61.5 ± 10.49 years); 41.3% of them had VI and 3.6% were blind. Cataracts (76.8%) and DR (7.1%) were the common causes of compensated VI and blindness. Risk factors that were associated with VI and blindness were age, monthly income, compliance with losing weight and physical activity.

**Conclusion:**

Findings suggest that lifestyle intervention and appropriate eyecare programmes may reduce VI and blindness in this population.

## Abstrait

**Facteurs de risques de déficiences visuelles et de cécité chez les adultes diabétiques noirs sud-africains recevant un traitement dans les établissements hospitaliers gouvernementaux du District de Mopani**.

**Historique:**

Le Diabète Mellitus (DM) est une maladie systémique commune chez les Sud-africains noirs. Cela peut entrainer la rétinopathie diabétique (DR), une cause commune de déficience visuelle (VI) et de cécité. La DR peut augmenter considérablement la prévalence de VI et de cécité.

**Objectifs:**

Evaluer les facteurs à risque de VI et de cécité dans la population de diabétiques noirs sud-africains âgés de ≥ 40 ans.

**Cadre:**

L’étude a été menée dans sept établissements de santé du gouvernement (deux hôpitaux, quatre cliniques et un centre de santé) dans le District de Mopani, dans la province du Limpopo, en Afrique du Sud.

**Méthode:**

C’était une étude quantitative transversale au sein d'un établissement. On a utilisé des interviews structurées pour obtenir des informations, comprenant des profils sociodémographiques, des connaissances sur le DM et ses complications oculaires, la présence d'hypertension et l'accès aux services de santé. Ensuite, les participants ont été examinés pour détecter les problèmes de vision et la cécité à l'aide d'un appareil auto-réfracteur, un disque à trou, un ophtalmoscope, et un tableau logMAR d'acuité visuelle (VA). En outre, on a pris les mesures anthropométriques (taille, poids et tour de taille). On a examiné statistiquement le rapport entre 31 facteurs de risques et le VI ainsi que la cécité.

**Résultats:**

Les participants (*N* = 225) comprenaient 161 femmes et 64 hommes âgés de 40 à 90 ans (moyen = 61.5 ± 10.49 ans), 41.3% d'entre eux avaient le VI et 3.6% étaient aveugles. La cataracte (76.8%) et le DR (7.1%) étaient les causes communes de VI compensé et de cécité. Les facteurs de risques associés au VI et à la cécité étaient l’âge, les revenus mensuels, l'acceptation de perdre du poids et l'activité physique.

**Conclusion:**

les résultats suggèrent que le changement de mode vie et les programmes appropriés de soins visuels peut réduire le VI et la cécité dans cette population.

## Introduction

Diabetes mellitus (DM) is a common metabolic disorder characterised by sustained hyperglycaemia of varying severity secondary to lack and/or diminished efficacy of endogenous insulin.^1^ Based on aetiology DM can be classified into type 1 (insulin-dependent) DM, type 2 (non-insulin-dependent) DM, gestational DM, and other specific types of DM.^2^ Type 1 DM (T1DM), which accounts for only 5% – 10% of all types of DM, results from a cellular-mediated auto-immune destruction of β-cells of the pancreas. This cell destruction leads to absolute insulin deficiency and dependence on exogenous insulin for survival.^3^ Type 2 DM (T2DM) accounts for about 90% – 95% of all types of DM and results from insulin resistance and relative insulin deficiency.^3^ T2DM can be controlled through healthy diet, participating in physical activities, losing excess weight and taking oral medication.^4^ Gestational DM occurs only during pregnancy and is a risk factor for T2DM after pregnancy.^5^ Other specific types of DM may be due to other causes such as genetic defects in β-cell function, insulin action, diseases of the pancreas, and drug-(such as HIV medication)or chemical-induced DM.^3^

Globally the number of people with DM is projected to double between the years 2000 and 2030, because of population aging, unhealthy diet, obesity and sedentary lifestyles.^6^ According to the International Diabetes Federation^7^ the number of people with DM in the sub-Saharan African region is projected to increase from 14.7million in 2011 to 28 million by 2030. In South Africa the number of adults (20–79 years) with DM is projected to increase from 1.9 million in 2011 to 2.5 million in 2030, with at least 78% of those with the condition being undiagnosed.^7^ The long-term effects of DM include, amongst others, development of diabetic retinopathy (DR), a common cause of visual impairment (VI) and blindness amongst adults aged 20 to 65 years.^7^ Some authors^8, 9^ have reported that people with DM are more likely to be visually impaired than those without it.

The World Health Organization (WHO)^10^ defines a risk factor as any attribute, characteristic or exposure of an individual that increases the likelihood of developing a disease or injury. Several demographic and socio-economic risk factors have been reported to be significantly associated with VI and blindness in the general population, including age,^9^ gender,^9^ educational and economic status.^8, 9^ Sociodemographic profiles such as age,^11, 12, 13, 14^ female gender,^12, 14^ low level of education,^8, 15^ unemployment^12^ and low income^9^ have been reported to be positively associated with VI and blindness amongst DM patients. Further, types of DM,^16, 17, 18^ insulin treatment^12, 14^ and hypertension^13, 14^ have been found to be positively associated with VI and blindness. A negative association has been found between DR and physical activity, weight loss and special diet compliance.^19^ Smoking status^20^ and anthropometric features^21^ such as high waist circumference (WC) and high body mass index (BMI) have also been reported to be positively associated with DR.

Knowledge about DM and its complications has an effect on compliance with treatment and successful management of the disease.^22^ Regular visits to medical clinics have been identified as a proxy indicator of better primary prevention of DM eye complications, and participants with irregular visits were found to be at higher risk of VI and blindness than those with regular visits.^23^

In a study conducted on 795 Taiwanese patients^24^ the average duration from a state of absence of signs of DR to background DR and blindness was approximately 10 years and 23 years respectively, suggesting that longer duration could be a risk factor for VI and blindness. Early detection and treatment of DR may lead to 60% reduction in DR progression from preproliferative diabetic retinopathy (PPDR) to proliferative diabetic retinopathy (PDR), and 57% reduction in the progression from PDR to blindness.^24^

No previous literature report could be found on the risk factors for VI and blindness amongst people with DM in South Africa.^25^ Such a report could be useful to the health authorities in planning for the prevention and elimination of modifiable risk factors associated with VI and blindness amongst people with DM. Therefore the purpose for this article was to investigate the risk factors for VI and blindness amongst black South Africans with DM aged ≥ 40 years who were receiving treatment at Government healthcare facilities in Mopani District.

## Research methods and design

### Study design

This was a cross-sectional health facility-based quantitative study.

### Setting

The study was conducted in seven Government health facilities in Mopani District, which included four clinics (Carlota, Dan, Ga-kgapane and Tzaneen), two hospitals (Ga-kgapane and Letaba), and one health centre (Nkowankowa). During the period of this study (May–December 2011) the total number of black South Africans with DM recorded in the chronic diseases registers at these seven health facilities was 721. Of this number, 25 (3.5%) were < 40 years of age, which included 15 women and 10 men. The 696 (96.5%) others were ≥ 40 years of age and included 475 (68.2%) women and 221 (31.8%) men.

### Study population and sampling strategy

The population was black South Africans of both sexes with DM, aged ≥ 40 years and receiving diabetes treatment from the targeted health facilities. The inclusion criteria included being black South African with DM, aged ≥ 40 years, willing to participate and signing the consent form. Convenience sampling was used to select participants from the targeted facilities. This method is non-probability sampling, which involves the use of the most conveniently available people as study participants. The advantages of this method include ease of recruitment, easier monitoring and follow-up, generally good response rates and retention of sample members.^26^ All of the patients who came to receive DM treatment and who satisfied the inclusion criteria were requested to participate. The plan was to have an equal number of participants from each health facility, but that was not possible because the number of DM patients varied significantly from one facility to another. Based on an estimate of 15% prevalence of VI and blindness amongst the target population, the calculated sample size was 195:

1 $$N=\frac{Z^{2*}\left[P\left(1-P\right)\right]}{D^{2}}$$

where *N* = sample size required; 95% confidence level is *z*^2^ (two-tail) = 1.96,

prevalence of VI and blindness is *P* = 0.15, and acceptable error is *D*^2^ = 0.0025.

Whilst the calculated sample size requirement was 195, 225 participants were included in this study.

### Data collection

First structured interviews were used to collect information which included sociodemographic profiles, knowledge of DM and its ocular complications, hypertension, smoking habits as well as accessibility to health facilities. Secondly, anthropometric measurements (height, waist and weight) were taken using a tape measure and bathroom scale. BMI (kg/m^2^) was calculated as weight (kg) divided by height in square metres (m^2^).^27^ BMI was graded according to the WHO classification,^27^ where ‘normal’ refers to a BMI of < 25 kg/m^2^, being ‘overweight’ to a BMI of 25–29 kg/m^2^, and ‘obese’ to a BMI of ≥ 30 kg/m^2^. WC was measured by locating the upper hip bone of the participant and placing a measuring tape around the abdomen, ensuring that the tape was snug, but did not compress their skin and was parallel to the floor. The participant was asked to relax and exhale, and then the measurement was taken.^28^ Three readings were taken for each of the anthropometric measurements and the average of each was recorded.

Thirdly, refractive errors were neutralised with optical correction from autorefraction values and visual acuity (VA) was measured with the participants wearing the optical correction. In cases where VA could not improve to better than 6/9.5, a pinhole disc was placed over the optical correction to rule out any residual uncorrected refractive error. An ophthalmoscope was used to determine the cause of VI or blindness. Thirteen participants were referred to the hospital ophthalmologist for a second opinion on diagnosis. All participants with treatable eye conditions and refractive error were referred to the ophthalmic nurses for treatment and/or for referral. Only the primary cause of VI was recorded. Where there were two or more primary disorders equally contributing to the visual loss, the WHO convention was followed, which is to record the cause that is easiest to treat and prevent.^29^ VI was defined as VA of worse than 6/9.5, but equal to or better than 3/60, and blindness was defined as VA of worse than 3/60 to no light perception (NLP). Therefore, for the purposes of this study, VI and blindness refer to VA from worse than 6/9.5 to NLP. This definition was adapted from the definition of VI and blindness by the WHO.^30^

Finally, 31 risk factors were statistically tested for association with VI and blindness.

### Data analysis

Data were analysed using the Statistical Analysis System (SAS) version 9.2 software package. Chi-square tests of association as well as logistical regression were performed, and odds ratios were calculated and interpreted. The level of significance was set at 0.05. There was no significant difference between the means of the VAs of the two eyes (*t*-value = 0.47, df = 224, *p* = 0.638). Therefore data from the right eyes only were used to study the associations. This is in keeping with usual practice for statistical research, where one eye from each subject is used to avoid a lack of independence of data.^31^

### Ethical considerations

This study was approved by the Health Studies Research and Ethics Committee of the University of South Africa (Project number: 0729-138-8), and permissions were obtained from the relevant authorities before commencement of the study. All the relevant ethics protocols were observed before, during and after the study.

## Results

**FIGURE 1:**
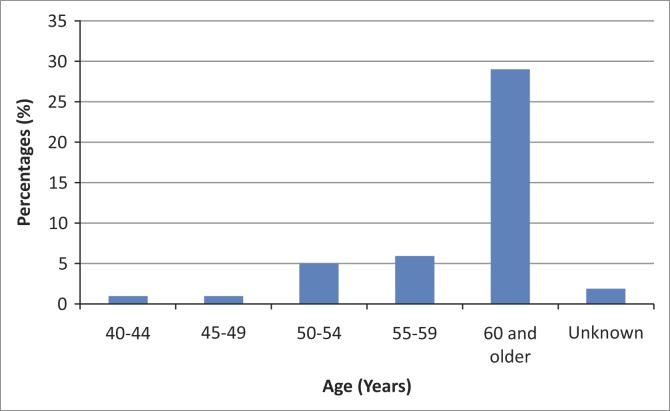
Prevalence of visual impairment and blindness by age of participants.

Of the 225 participants, 161 (71.6%) were women and 64 (28.9%) were men. Their ages ranged from 40 to 90 years (mean 61.5 ± 10.49 years), and 41.3% had VI and 3.6% were blind. Cataracts (76.8%) and DR (7.1%) were the most common causes of compensated VI and blindness. The prevalence of VI and blindness ranged from 0.9% amongst those aged 40–44 years to 29.3% amongst those aged ≥ 60 years (Figure1. There was a significant association between age and VI and blindness (df = 4, *χ*^2^ = 11.2, *p* = 0.02).

Table 1 shows the demographic risk factors for VI and blindness that were examined, and results of chi-square tests. Only age and monthly income were statistically associated with VI and blindness (Table 1). The prevalence of VI and blindness was higher (33.4%) amongst those earning ≤ R2000 a month than amongst those earning > R2000 (4%) (Table 2). In addition, the probability of those earning ≤ R2000 a month being visually impaired is 3.14 times that of those earning > R2000. Monthly income was statistically associated with VI and blindness (df = 4, *χ*^2^ = 14.0, *p* = 0.007).

**TABLE 1:** Demographic and anthropometric risk factors examined.

Risk factors	VI/blind			Variables	
	
	Df	*χ*^2^	*P-*value	*N*	%
Age	4	11.2	0.02*	225	100
Monthly income	4	14.0	0.007*	225	100
Gender	1	0.1	0.799	224	99.6
Marital status	3	3.8	0.285	225	100
Educational level	3	5.2	0.156	223	99.1
Residence	1	1.1	0.299	219	97.3
BMI	2	0.0	0.994	200	88.9
WC	5	0.6	0.990	224	99.6

* Only age and monthly income were statistically associated with VI and blindness.

VI, visual impairment; BMI, body mass index; WC, waist circumference.

**TABLE 2:** Prevalence and distribution of visual status by monthly income.

Monthly income	VI/blind		Not VI		Total	
		
	*N*	%	*N*	%	*N*	%
No income	17	7.6	17	7.6	34	15.1
≤ R500	4	1.8	11	4.9	15	6.7
R501 – R1000	4	1.8	11	4.9	15	6.7
R1001 – R2000	67	29.8	58	25.8	125	55.6
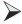 R2000	9	4	27	12	36	16.0
**Total**	**101**	**44.9**	**124**	**55.1**	**225**	**100**

VI, visual impairment.

For brevity, only statistics on risk factors that are statistically associated with VI and blindness are elaborated upon in this article; others are briefly presented. Gender was not statistically significant (*p* = 0.799). The prevalence of VI and blindness amongst those who were single, married, divorced and/or separated, and widowed was 51.6%, 41.1%, 42.9% and 61.2% respectively, and marital status was not associated with VI and blindness (*p* = 0.285).

Amongst the 57 participants with no formal education, 54.4% had VI and blindness. VI and blindness prevalence was higher amongst those with primary (19.3%) than those with tertiary (2.7%) education, but the difference was not statistically significant (*p* = 0.156). The prevalence amongst those living in rural and semi-urban areas was 43.9% and 51.6% respectively, but place of residence was not associated with VI and blindness (*p* = 0.299).

VI and blindness was highest (18.5%) amongst those who were obese and lowest (1%) amongst those who were underweight. However, there was no association between VI and blindness and BMI (*p* = 0.994). The prevalence amongst male participants with WC > 94 cm was 46% and 36.4% amongst those with WC ≤ 94 cm. Amongst the female participants, the prevalence was 53.9% amongst those with WC > 80 cm, and there was no female with a WC of ≤ 80 cm. There was no association with WC (*p* = 0.990).

Table 3 shows the clinical risk factors for VI and blindness and the results of chi-square tests. Only physical activity and compliance with losing weight were statistically associated with VI and blindness.

**TABLE 3:** Clinically related risk factors examined.

Risk factors	VI/blind			Variables	
	
	df	χ^2^	*P-*value	*N*	%
Physical activity	1	6.0	0.014	207	92.0
Weight loss compliance	2	9.4	0.009	117	52
Duration of DM	3	1.8	0.614	225	100
Knowledge of types of DM	2	0.2	0.920	222	98.7
DM type	1	0.1	0.714	222	98.7
Special diet	1	0.1	0.705	220	97.8
Losing weight	1	1.0	0.314	204	90.7
Special diet compliance	1	0.5	0.468	213	94.7
Physical activity compliance	2	2.6	0.278	145	64.4
Date of last DM check-up	1	1.4	0.238	224	99.6
DM family history	2	2.4	0.305	220	97.8
Knowledge that DM can cause VI	1	0.8	0.359	224	99.6
Knowledge that DM can cause DR	1	0.3	0.601	223	99.1
Knowledge that DM can cause glaucoma	1	0.0	0.973	224	99.6
Eye examination history	1	1.3	0.255	217	96.4
Last eye examination	4	9.1	0.059	119	52.9
Family members with VI	2	0.3	0.859	169	75.1
Date for last blood pressure check-up	1	0.5	0.469	225	100
Hypertension	1	0.2	0.652	217	96.4
Hypertension treatment	1	0.5	0.463	177	78.7
Smoking status	3	4.4	0.214	213	94.7
Age when started smoking	4	2.4	0.660	225	100
Accessibility to health services	4	2.2	0.693	219	97.3

VI, visual impairment; DM, Diabetes mellitus; DR, diabetic retinopathy.

The prevalence of VI and blindness amongst those who engaged in physical activity was 22.7% and it was 20.3% amongst those who did not; there was a significant association between VI and blindness and physical activity (df = 1, *χ*^2^ = 6, *p* = 0.014, odds ratio 0.51). The prevalence of VI and blindness was 7.7%, 17.1% and 16.2% respectively amongst those who reported engaging in weight loss activities always, not always and those who did not. There was a significant association between compliance with losing weight and VI and blindness (df = 2, *χ*^2^ = 9.4, *p* = 0.009).

Prevalence of VI and blindness was highest (16.4%) amongst those who had had DM diagnosed for < 5 years and lowest (1.3%) amongst those who had had it for longer periods (16–20 years). However, this association was not significant (p = 0.614). Prevalence of VI and blindness was 33.7% amongst participants who were not aware of the existence of different types of DM, 6.6% and 7.6% amongst those who knew one type and two types respectively. The prevalence amongst type 1 and type 2 patients was 4.1% and 40.5% respectively. Both knowledge and the types of DM were not associated with VI and blindness (*p* = 0.920 and *p* = 0.714 respectively).

Prevalence of VI and blindness was highest (21.9%) amongst those who had an eye examination within a period of one year or more before the study, and the lowest (0.8%) amongst those who were examined less than one month earlier (Table 4). There was no significant association between date of last eye examination and VI and blindness (df = 4, *χ*^2^ = 9.1, *p* = 0.059). The period of the last eye examination in relation to VI is shown in Table 4.

**TABLE 4:** Period since last eye examination in relation to visual status.

Period since last eye examination	VI/blind		Not VI/blind		Total	
		
	*N*	%	*N*	%	*N*	%
< 1 week	0	-	0	-	0	-
< 1 month	1	0.8	12	10	13	10.9
< 6 month	11	9.2	8	6.7	19	16
< 1 year	8	6.7	8	6.7	16	13.5
≥ 1 year	26	21.9	28	23.5	54	45.4
Unknown	7	5.9	10	8.4	17	14.3
**Total**	**53**	**44.5**	**66**	**55.5**	**119**	**100**

VI, visual impairment.

Prevalence of VI and blindness amongst participants with and without hypertension was 35.5% and 8.6% respectively; however, the association was not significant (*p* = 0.652). Most (80.6%) of the participants had never smoked cigars, cigarettes or a pipe. Prevalence amongst those who never smoked, smoked occasionally, smoked regularly and always smoked was 37.1%, 3.3%, 0% and 5.2% respectively. There was no association between VI and blindness and smoking status (*p* = 0.214). The prevalence amongst those who walked < 30 minutes and for > 1 hour to the health facility was 16% and 3.2% respectively, with no significant association with accessibility (*p* = 0.693).

When the Proc Logistic of the SAS was used to fit models of all the above variables, only monthly income (df = 4, χ2 = 10.75, *p* = 0.03) and physical activity (df = 2, χ2 = 14.96, *p* = 0.00) remained significantly associated with VI and blindness. Age and compliance with losing weight were not associated in the multivariate analysis.

## Discussion

VI and blindness due to DM is largely preventable through early detection, monitoring and management of diabetic eye diseases.^24^ Risk factors that were individually associated with VI and blindness in this study were age, monthly income and compliance with losing weight and physical activity. However, only monthly income and physical activity remained significantly associated following multivariate analysis. Some of the risk factors reported in this article are not necessarily specific for persons with DM alone, but are also risk factors for VI and blindness in the general population.

The positive association of increasing age with VI and blindness found in this study is in agreement with findings from several previous studies.^12, 13, 14, 15, 32, 33^ The probable explanation for the association is that many blinding eye diseases, such as cataract, glaucoma and DR, are age-related. The fact that a larger percentage of the participants were ≥ 60 years of age may explain the association between age and VI and blindness in this study. It is therefore important that people in this age group have regular eye examinations so that conditions (DM- and non-DM-related) that may result in VI and blindness may be detected early.

In addition, low monthly income was significantly associated with VI and blindness. This agrees with the findings of a previous study^9^ that low income earners in the general population were twice as likely to have VI as those with higher income. Low socio-economic status^14^ has also been reported to be significantly associated with increased risk of VI. However, findings in this study disagree with those of another study^32^ where no association was reported.

That the prevalence of VI and blindness was high amongst the participants with a monthly income of ≤ R2000 may be explained by the fact that many of them were elderly and receiving the Government old-age pension of about R1200. They therefore may not be able to afford eyecare services. A contributory factor may be poor access to cataract surgery and affordable spectacles provided by Limpopo province; these are available only at Elim and Mankweng Hospitals, which are about 150km from the site of this study. Some studies^12, 15, 24^ found a positive association between female gender and VI and blindness. However, in agreement with previous reports,^13, 34^ no association between these variables was found in this study.

Previous authors^8, 9, 12, 35^ have reported that the prevalence of VI tends to decrease significantly with increasing level of education. This is because people with higher levels of education are more likely than those with low levels to seek medical intervention before they are visually impaired or blind, as they are better informed about the related risk factors. In addition, persons with tertiary education are likely to have higher income than those with primary education, and could therefore afford spectacles and cataract surgery.^35^ Further, a higher level of education is associated with a greater likelihood of seeking eyecare services, better knowledge and more reasonable health-seeking behaviour.^35^ Contrary to those reports, there was no association between educational qualification and VI and blindness in this study. This was the case despite the fact that the prevalence of VI and blindness was lower amongst those with tertiary education than those with primary education.

The higher prevalence of VI and blindness amongst those living in rural areas compared with those living in other places may be because most (68.9%) of the participants in this study were from rural areas. Another possible explanation is that there is a lack or shortage of eyecare services in the rural areas, a common situation in South Africa. In addition, services provided in the urban areas are often better than those that are available in the rural areas.^36^ Poor economic status, lack of transportation, low literacy level, lack of awareness and traditional beliefs of rural dwellers have been reported to be responsible for underutilisation of available eyecare services.^36^ It has been recommended that eyecare services for disadvantaged communities should include education and eye health promotion as preventive measures.^37^

Although a previous study^13^ found that higher BMI was positively associated with VI and blindness, others^33, 38, 39^ found low BMI to be positively associated with VI and blindness. In this study there was no significant association between VI and BMI, although VI and blindness was more common amongst obese participants (BMI ≥ 30 kg/m^2^) than those who were not obese. This may be attributed to the fact that most of the participants in this study were obese. This finding is in agreement with those of other studies^11, 14^ which did not find any significant association.

A WC of > 94 cm for men or > 80 cm for women has been reported to be associated with an increased risk of diseases of lifestyle^27^ such as DM and hypertension. A WC of > 102 cm for men or > 88 cm for women has been reported to be associated with a substantially increased risk of diseases of lifestyle.^27^ Although the prevalence of VI and blindness amongst the participants in this study was higher in those with a WC of > 94 cm and > 80 cm, for 46% versus 36.4% (for men) and 53.9% versus 0% (for women) respectively, there was no association between VI and blindness and WC. The prevalence of VI and blindness was lowest amongst those who reported trying to lose weight. In addition, the risk of being visually impaired amongst those who engaged in physical activity is 0.51 that of those who did not. The association between losing weight as well as physical activity with VI can be explained by the fact that those factors help in glycaemic control, which is important in the control of DM and therefore prevention of diabetic eye diseases that can result in VI and blindness.

Several studies^12, 32, 33, 34, 35^ have found longer duration of DM to be positively associated with high prevalence of VI. This is expected because the severity of DR increases with duration of DM. However, there was no association between duration of DM and development of VI in this study. This could be due to the fact that a larger percentage (39.6%) of the participants was diagnosed with DM within < 5 years of this study. Further, more than half (54.7%) of the participants were aged ≥ 60 years, suggesting that their VI might be due to age-related eye diseases.

The higher prevalence of VI and blindness amongst the T2DM patients than amongst the T1DM patients in this study agrees with findings from other studies.^15, 17^ However, there was no association between the types of DM and VI and blindness in this study. An obvious explanation for the higher prevalence of VI and blindness in the T2DM category is the greater number of T2DM participants (90.1%). In addition, T2DM patients are generally older than T1DM patients, which suggests that some of the VI and blindness may be age-related.

The non-association of prevalence of VI and blindness with smoking in this study agrees with the findings of previous studies,^12, 34^ but disagrees with that of another study^22^ in which smoking was associated with DR. This may be because most (80.6%) of participants in this study had never smoked cigars, cigarettes, or a tobacco pipe. The higher prevalence of VI and blindness amongst participants who lived closer to the facilities rather than far away may be attributed to the fact that there were more participants in the former group than in the latter. Accessibility to health facilities was not associated with VI and blindness (*p* = 0.693). The prevalence of VI and blindness was higher amongst those last examined > 1 year ago compared to those last examined < 1 year ago (*p* = 0.059). More participants in the former group probably had undiagnosed or untreated eye conditions prior to the research study. This agrees with the views of other authors^18^ who reported that a regular visit to medical clinics was a proxy indicator of better primary prevention of DM eye complications.

### Strengths and limitations

This study is the first to describe the risk factors for VI and blindness amongst black South Africans with DM in a predominantly rural district of Limpopo province, and therefore provides valuable data on these risk factors in the community. The fact that this study was conducted in Government health facilities and not population-based could have introduced a health-seeking bias. The larger proportion of older, unemployed and poor participants, and there being more women than men in this study, could also introduce some bias. This could be due to the fact that those who were employed (mostly men) might have been at work when the study was conducted, or it might mean that men do not survive DM as well as women. In addition, individuals in formal employment (mostly men) tend to have medical aid schemes and therefore use private health services. It is acknowledged that the study population is not representative of the entire population of persons with DM in the district.

### Recommendations

Based on the findings in this study it is recommended that a qualitative study be conducted to shed light on some of the findings amongst rural Africans with DM in Limpopo province. For example, could there be personal, social or cultural barriers to exercise, compliance to a special diet and use of eyecare services in this population? If such a study reveals no barriers, it would then be recommended that efficient and targeted lifestyle intervention programmes that focus on physical activity and losing weight be initiated to reduce the modifiable risk factors for VI and blindness in this population. An awareness-raising campaign should also be established to educate this population about control of DM and early detection of DR, as well as other causes of VI amongst diabetics, including refractive errors, cataract and glaucoma. In addition, refraction and cataract surgical services should be made available and accessible to this and other rural populations of Limpopo province.

## Conclusion

This study demonstrated that age, monthly income, compliance with losing weight and physical activity were individually associated with VI and blindness; however, only monthly income and physical activity remained significantly associated following the multivariate analysis. Findings in this study may be useful to health authorities in planning for prevention of VI and blindness, as well as DM intervention programmes in the province.
